# Transcriptional profiling of the response to starvation and fattening reveals differential regulation of autophagy genes in mammals

**DOI:** 10.1098/rspb.2023.0407

**Published:** 2023-03-29

**Authors:** Margarita Galves, Michal Sperber, Fatima Amer-Sarsour, Ran Elkon, Avraham Ashkenazi

**Affiliations:** ^1^ Department of Cell and Developmental Biology, Sackler Faculty of Medicine, Tel Aviv University, 6997801 Tel Aviv, Israel; ^2^ Department of Human Molecular Genetics and Biochemistry, Sackler Faculty of Medicine, Tel Aviv University, 6997801 Tel Aviv, Israel; ^3^ Sagol School of Neuroscience, Tel Aviv University, 6997801 Tel Aviv, Israel

**Keywords:** transcriptional networks, autophagy, nutrient deprivation, high-fat diet

## Abstract

Nutrient deprivation (starvation) induced by fasting and hypercaloric regimens are stress factors that can influence cell and tissue homeostasis in mammals. One of the key cellular responses to changes in nutrient availability is the cell survival pathway autophagy. While there has been much research into the protein networks regulating autophagy, less is known about the gene expression networks involved in this fundamental process. Here, we applied a network algorithm designed to analyse omics datasets, to identify sub-networks that are enriched for induced genes in response to starvation. This enabled us to identify two prominent active modules, one composed of key stress-induced transcription factors, including members of the Jun, Fos and ATF families, and the other comprising autophagosome sub-network genes, including ULK1. The results were validated in the brain, liver and muscle of fasting mice. Moreover, differential expression analysis of autophagy genes in the brain, liver and muscle of high-fat diet-exposed mice showed significant suppression of GABARAPL1 in the liver. Finally, our data provide a resource that may facilitate the future identification of regulators of autophagy.

## 1. Introduction

Macroautophagy (henceforth autophagy) is a degradation process that mediates the removal of long-lived proteins and organelles within cells [1–4]. This is accomplished by double-membraned autophagosomes, which surround and engulf portions of cytoplasm that are targeted for degradation and recycling in the lysosomes [2,5,6]. The formation of autophagosomes requires nucleation, elongation and closure, and is governed by conserved autophagy-related (ATG) proteins [7]. These ATG proteins are also involved in the maturation of autophagosomes and their fusion with lysosomes [2,7,8]. The basic function of autophagy, which is a conserved feature of eukaryotes, is to protect cells against nutrient deprivation (termed starvation in cells or fasting *in vivo*) [9,10], and in this context, autophagy activation is particularly important for survival during neonatal starvation [11]. By upregulating autophagy under these conditions, cells degrade macromolecules and organelles into their building blocks, which can then be re-used to build proteins and membranes, and as a source of energy [11–13].

In mammals, fasting induces autophagy in a variety of tissues, including brain, muscle and liver [9,14,15]. Many of the studies exploring the regulation of starvation-induced autophagy in mammalian cells have focused on changes in protein levels and post-translational modifications, such as protein phosphorylation and ubiquitination [14,16–21]. Examples include inhibition of the mammalian target of rapamycin complex 1 (mTORC1), activation of Unc-51-like autophagy activating kinase 1 (ULK1), and the activation of beclin 1 complexes [1,19,22,23].

In addition, a number of transcription factors, such as TFEB, the FOXO family of transcription factors, ZKSCAN3 and NFκB, have been identified as involved in autophagy regulation and ATG gene expression under conditions of nutrient deprivation [24–28]. TFEB and the FOXO family are positive regulators of autophagy, while ZKSCAN3 appears to negatively regulate the process [26,29]. However, how such transcriptional regulation affects messenger RNA (mRNA) expression in nutrient-deprived mammalian cells and tissues is not yet fully understood.

Here, we performed transcriptional profiling in human cells in order to identify biological processes associated with the response to starvation. For this purpose, we employed an algorithm called 'discovery of active modules in networks using omics' (DOMINO) that we recently designed for active module identification. DOMINO is able to detect active network modules with high confidence [30]. By using DOMINO, we identified two functional modules that were activated upon starvation: one composed of stress-induced transcription factors, and the other a specific ATG network related to ULK1, which was further examined in fasting mice. Moreover, since autophagy induction can protect from deleterious effects of high-fat diet in mice [31], we also explored whether specific autophagy genes were downregulated in tissues from fat mice. Our unbiased analysis identifies prominent gene networks in the transcriptional response to fasting and fattening in mammals.

## 2. Material and methods

### (a) Cell culture and treatment

HeLa cells were purchased from ATCC (CCL-2) and were maintained in a humidified incubator with 5% CO_2_ at 37°C in DMEM (01-052-1A, Biological Industries) supplemented with 10% heat-inactivated fetal bovine serum (FBS) (04-007-1A, Biological Industries), penicillin/ streptomycin (15240-062, Gibco) and l-glutamine (G7513, Sigma). The cells were authenticated by short tandem repeat (STR) profiling and were routinely tested for mycoplasma contamination. For nutrient starvation experiments, cells were washed with phosphate-buffered saline (PBS) and treated with HBSS (14025092, Gibco) for 4 h.

### (b) RNA extraction from cells

Total RNA was extracted from HeLa cells using TRIzol (009010233100, Bio Labs) according to the manufacturer's instructions. RNA (1–3 µg) was reverse-transcribed using RevertAid reverse transcriptase (K1621, Thermo Fisher) according to the manufacturer's instructions.

### (c) RNA-seq experiments

RNA-seq libraries were prepared using the mRNA-seq protocol of the Crown Genomics Institute of the Nancy and Stephen Grand Israel National Center for Personalized Medicine, Weizmann Institute of Science. Briefly, the polyA fraction (mRNA) was purified from 500 ng of total input RNA followed by fragmentation, and generation of double-stranded complementary DNA (cDNA). This was followed by Agencourt Ampure XP bead cleanup (Beckman Coulter), end repair, A base addition, adapter ligation and PCR amplification. Libraries were quantified by Qubit (Thermo Fisher Scientific) and TapeStation (Agilent) and sequenced with a Nextseq instrument (Illumina) using a 75 cycle high output kit, allocating 20 million reads per sample (single-read sequencing).

### (d) RNA-seq analysis

Alignment and read counting per gene were performed using STAR [32], the hg38 human reference genome and GENCODE gene annotations. Read counts were converted to counts per million (CPM). Overall, 13 008 genes could be detected in our dataset. Differential expression was analysed using DESeq2 [33], according to three criteria: false discovery rate (FDR) < 1%; fold change > 2.5; and a consistency in the direction of the expression change over all replicates [34]. Gene ontology (GO) enrichment used the clusterProfiler package [35] with the parameters: ontology, biological process; *p*-value adjustment method, FDR; *p*-value cutoff, 1%; adjusted *p*-value cutoff, 5%). Heatmaps were generated using Heatmapper [36]. Raw fastq files and processed data were uploaded to GEO (accession code GSE211066). For the analysis of high-fat diet in mice, we obtained RNA-seq data of liver, skeletal muscle and brain from the GEO repository: accession codes GSE88818, GSE97718, GSE179711 [37–39].

### (e) Fasting experiments in mice

All mice experiments were approved by the Institutional Animal Care and Use Committee (IACUC) of Tel Aviv University. Mice used in this study are from the C57BL/6J background, for which suitable fasting-induced autophagy protocols have been described previously [14]. In brief, 7–8-week-old mice were food-deprived with free access to water. A period of 22 h fasting was used for experiments involving gastrocnemius muscle and liver extraction, with an extended period of 44 h for experiments involving brain extraction. After 22 h of food deprivation, the mice were allowed free access to food for 2 h, after which they were transferred to clean cages for an additional 22 h fast.

### (f) RNA extraction from mouse tissue and qRT-PCR

Selected tissues harvested from fed and fasted mice were washed with cold PBS containing calcium and magnesium and were then lysed with RLT buffer from the RNeasy Qiagen mini kit (cat. no. 74004) and homogenized with a TissueLyser II instrument (Qiagen) set at 30 strokes s^−1^ for 2 min. The homogenized tissue was centrifuged at 12 000*g* at 4°C for 15 min (brain and liver samples were centrifuged twice to obtain clearer supernatant). The supernatant was transferred to new tubes and RNA was extracted with TRIzol reagent (Invitrogen) as recommended by the manufacturer. The concentration and purity were determined on an ND1000 NanoDrop spectrophotometer (NanoDropTechnologies) and RNA (1–3 µg) was reverse-transcribed using RevertAid reverse transcriptase (K1621, Thermo Fisher Scientific) according to the manufacturer's instructions. The cDNA was subjected to qPCR using a SYBR Green mix buffer (PB10.22-02, PCR Biosystems) in a total of 10 µl reaction volume. Triplicate qRT-PCR samples were prepared using the 2× PCRBIO HS Taq Mix Red, and were amplified in a StepOnePlus instrument (Thermo Fisher). The expression level of each mRNA was normalized to β-actin, and the ΔΔ*C*_t_ method was used to calculate the level of normalized transcript. The primers used in the qRT-PCR were: β**-Actin-F**: CCTGAACCCTAAGGCCAACC; **β-Actin-R**: ATGGCGTGAGGGAGAGCATA; **ATG14-F**: TCACAGGCCCGTGGATTAGC; **ATG14-R**: CGCCACAGAACTCGCTGTTG; **ULK1-F**: ACGAAAACATCGTGGCGCTG; **ULK1-R**: GTGTGCGCATAGTGTGCAGG; **UVRAG-F**: GGCTACTACGGTGCTCCCTG; **UVRAG-R**: TGGGCTCTATGAAGCCGCAG.

### (g) Statistical analysis

Statistical analyses of the mouse experiments used GraphPad Prism software v. 9 and Student's *t*-test (two-tailed) where *p* < 0.05 was considered statistically significant. The statistical method used for analysis of RNA-seq data has been described in §2d.

## 3. Results

### (a) Transcriptional networks in the human cell response to starvation

**Figure 1.**
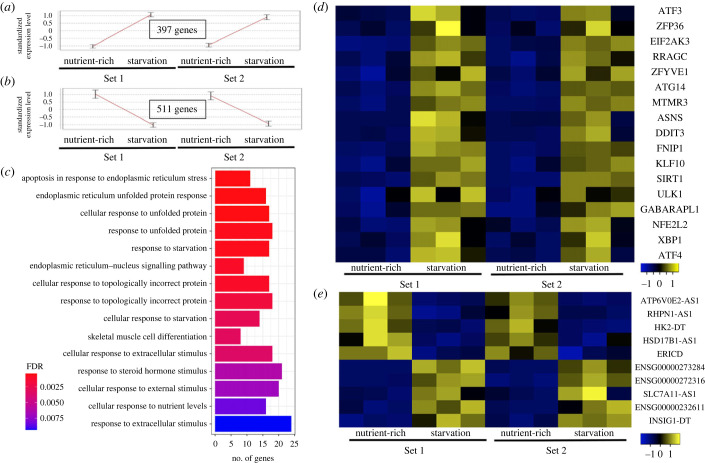
Transcriptional responses to starvation. (*a*,*b*) Expression pattern of the genes that were significantly induced (*a*) or repressed (*b*) in response to nutrient deprivation in HeLa cells. The expression level of each gene across the probed samples was standardized to mean = 0 and s.d. = 1 to focus on expression pattern (rather than on absolute levels). The curves show the mean pattern of the standardized expression levels, and error bars show ±1 s.d. (*c*) Enriched GO categories among the set of 397 genes induced by starvation (*p*-adjusted, represented by reddish colour scale, are the FDR-corrected *p*-values). (*d*) Consistent induction of known ‘starvation-genes' observed in our cellular model upon nutrient deprivation. (*e*) Top ranked induced and repressed long non-coding RNA (lncRNA) differentially expressed genes. Results were obtained from the analysis of the transcriptional responses to starvation in HeLa cells treated with nutrient-deprived medium for 4 h and control cells grown in nutrient-rich medium. Two independent batches with triplicate samples of each biological condition are shown.

We applied RNA-seq analysis to HeLa cells treated with nutrient-deprived medium for 4 h and control cells grown in nutrient-rich medium in order to explore transcriptional responses to starvation in mammalian cells. This starvation period was selected to be long enough to detect changes in the transcriptome but to be short enough to avoid any interference from secondary regulators. Two independent batches with triplicate samples of each biological condition allowed us to detect the expression of more than 13 000 genes (electronic supplementary material, table S1). Differential expression analysis identified 397 genes that were significantly and consistently induced, and 511 genes that were significantly and consistently repressed by starvation (figure 1*a,b*) (90% of the induced genes are protein-coding, while 7.3% are non-coding, and 88.2% of the repressed genes are protein-coding, while 8.4% are non-coding). GO enrichment analysis revealed that the set of induced genes was markedly enriched for genes known to play key roles in apoptosis (SIRT1, BCL2L11, BBC3, PPP1R15A, DDIT3, HERPUD1 and CHAC1), and the responses to unfolded proteins and endoplasmic reticulum stress (CREBRF, YOD1, ERN1, ASNS, DDIT3, DNAJB9, HERPUD1, NFE2L2, WIPI1 and DERL3), as well as the cellular responses to starvation (including FNIP1, SIRT1, ATG14, DDIT3, ATF3, ATF4, XBP1, ULK1 and ZFP36; figure 1*c*,*d* and electronic supplementary material, table S2).

We also focused our analysis on long non-coding RNAs (lncRNAs) because the majority of lncRNAs are transcribed by RNA polymerase II and are similar in structure to mRNA, implicated in gene regulation [40]. Our differential expression analysis identified the 10 lncRNAs that were most strongly induced or repressed by starvation (figure 1*e*). Several genes are represented by their Ensembl IDs since they are novel transcripts with no gene names. Among the known genes, the coverage of the lncRNA HK2-DT is presented in electronic supplementary material, figure S1. Moreover, the lncRNA RHPN1-AS1 was downregulated in our analysis of starvation-induced autophagy, and the inhibition of RHPN1-AS1 was recently shown to induce autophagy in prostate cancer-derived cells [41].

These results demonstrate that our starvation protocol is relevant for studying the transcriptional regulation in autophagy induction, apoptosis and endoplasmic reticulum stress [9,42]. Interestingly, SIRT1, a member of the conserved sirtuin family of nicotinamide adenine dinucleotide (NAD^+^)-dependent deacetylases was detected in multiple GO categories. That SIRT1 was recently reported to be regulated by starvation and autophagy [43] provides further support for the relevance of our data for starvation-induced transcriptional programme investigation.

### (b) DOMINO identifies signalling patterns of stress-induced factors and autophagy

**Figure 2.**
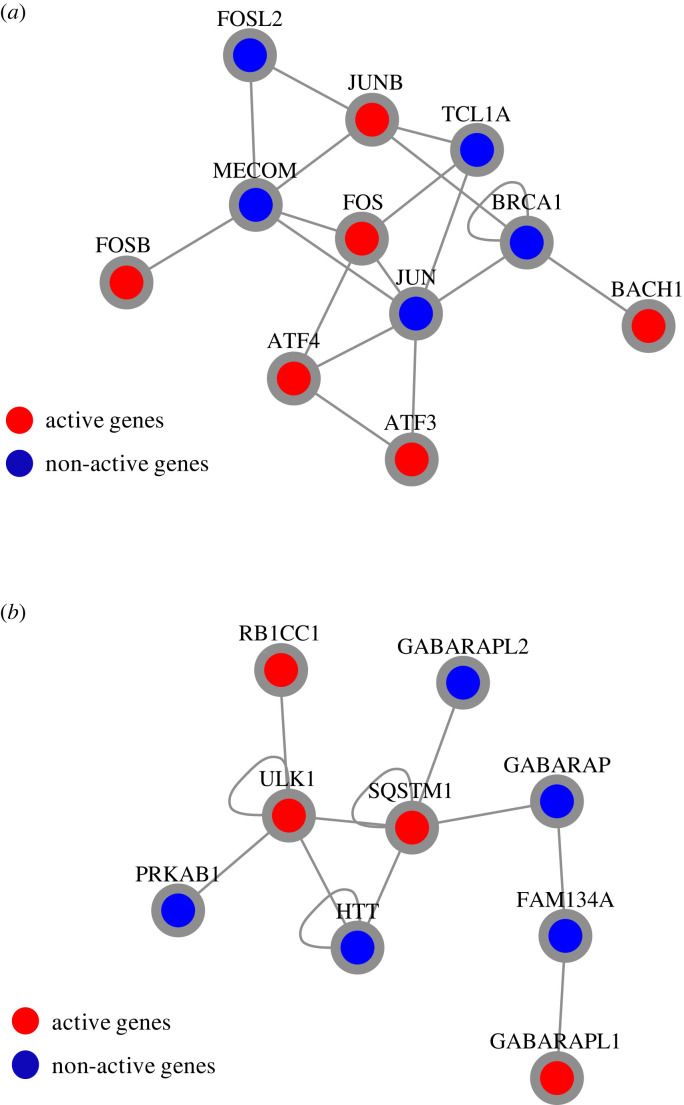
Active modules detected by DOMINO among the genes induced in the nutrient-deprived cells. Nodes represent genes/proteins and edges represent physical interactions among the protein products. Genes induced upon starvation in our dataset are coloured in red. Blue nodes were selected by the DOMINO algorithm to connect induced genes in dense sub-networks.

We used an integrated gene-expression and protein–protein interaction (PPI) network analysis to further characterize the biological endpoints of the transcriptional programme we detected as induced by starvation in our cellular model. For this purpose, we applied DOMINO [30], a network-based algorithm that we recently developed for analysis of omics datasets. Given an input omics dataset (e.g. differential gene expression scores) and a global PPI network, DOMINO searches the network for specific regions (neighbourhoods) that are enriched for highly scoring genes. Such enriched sub-networks are also called ‘hot functional modules' or ‘active modules'. DOMINO detected two prominent active modules for the genes induced in our dataset. The first module is composed of key stress-induced transcription factors, including members of the Jun, Fos and ATF families (figure 2*a* and electronic supplementary material, table S3). This is not unexpected since amino acid deprivation and endoplasmic reticulum stress are known to induce expression of multiple ATF mRNA species [44]. Notably, the second active module detected by DOMINO comprises fundamental ATGs involved in autophagosome formation (figure 2*b* and electronic supplementary material, table S4), including key regulators of autophagy, such as ULK1, RB1CC1, SQSTM1 and GABARAPL1. Interestingly, although the ULK1 complex is composed of four subunits (serine–threonine ULK1/2 kinase, ATG13, ATG101 and RB1CC1/FIP200 [45]), the DOMINO analysis identified only ULK1 and RB1CC1 as ‘active genes'. This suggests that there could be differential transcriptional regulation even between ATG genes related to the same functional autophagy-initiating complex. Moreover, although mammalian cells encode six known ATG8 orthologues, termed LC3A, LC3B, LC3C, GABARAP, GABARAPL1 and GABARAPL2, DOMINO selected only GABARAPL1 as an active gene of the starvation response. This is consistent with the emerging role of the GABARAP subfamily in both autophagosome formation and autophagosome–lysosome fusion [46,47].

**Figure 3.**
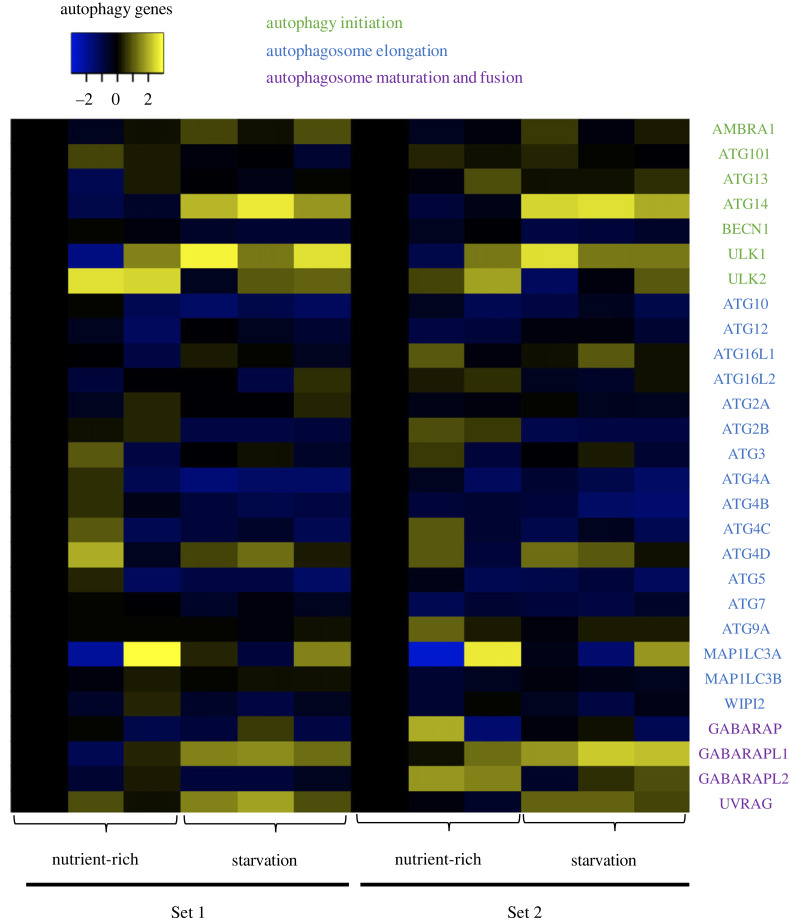
Selective induction of autophagy-related (ATG) genes upon starvation. The heatmap shows the transcriptional response of ATG genes, only a subset of which (e.g. ATG14, ULK1) showed an induction in gene expression in the nutrient-deprived cells (represented by yellowish colour scale).

These results provide an unbiased method to identify specific ATG components associated with the transcriptional starvation response. A further examination of a comprehensive set of ATG genes identified additional key regulator genes, including ATG14 and UVRAG, that were induced upon starvation (figure 3). ATG14 and UVRAG form two distinct complexes with beclin-1 that regulate VPS34 synthesis of phosphatidylinositol-3-phosphate and consequently affect autophagosome formation or maturation, respectively [23]. Notably, our analysis revealed that beclin-1 is less affected by the starvation-induced cellular transcriptional programme than ATG14 and UVRAG.

### (c) Validation of the DOMINO-detected module in fasting mice

**Figure 4.**
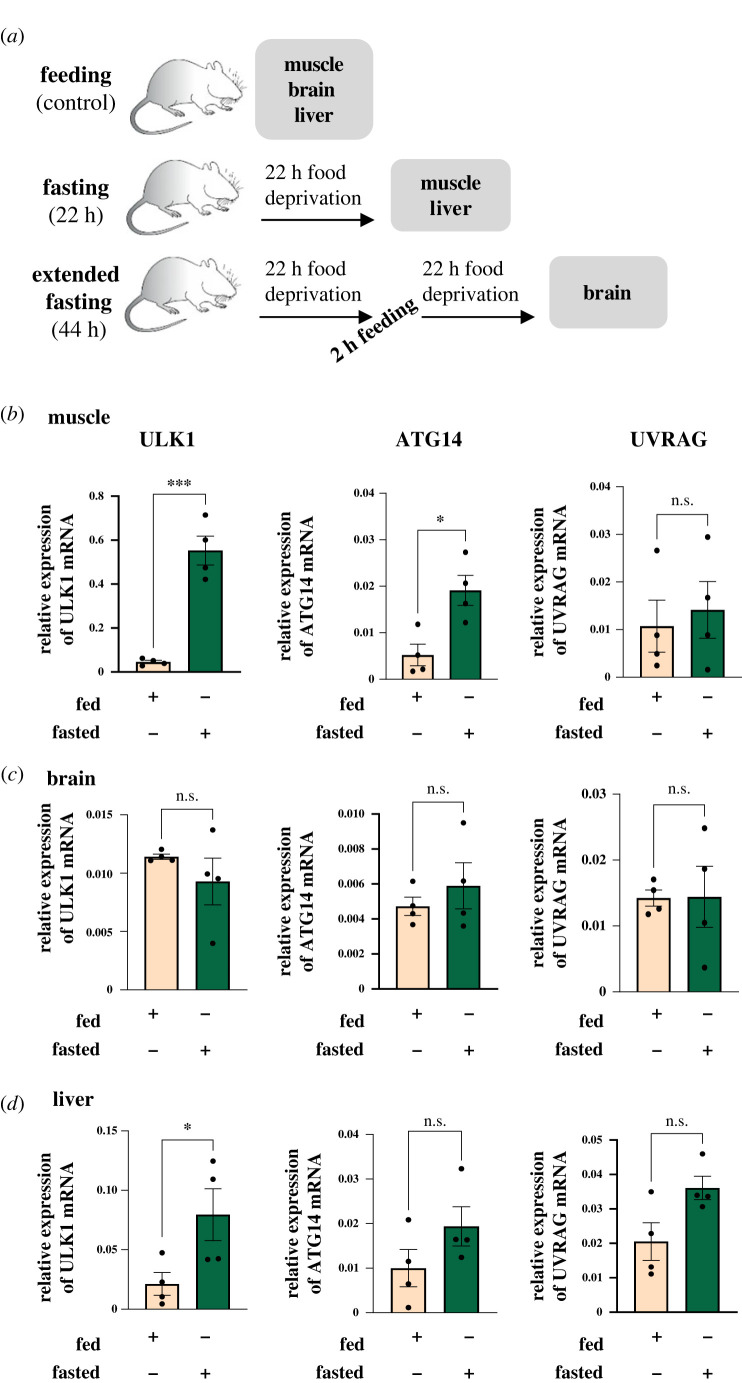
Autophagy-related gene expression in fed and fasting mice. (*a*) Description of the fasting regimen used in this study for C57BL/6 J mice. (*b*–*d*) Quantification of ULK1, ATG14 and UVRAG mRNA levels in different tissues from fed and fasted mice by qRT-PCR. Fold change was calculated after normalization to β-actin using the ΔΔ*C*_t_ method. (*b*) Muscle, (*c*) brain and (*d*) liver. Average and s.e.m. are presented (*n* = 4 mice per group). *p < 0.05, ***p < 0.001, n.s., not significant. Two-tailed Student's *t*-test.

The physiological relevance of our findings was validated by fasting experiments in mice. Autophagy was induced in mice by established protocols that we and others have shown to cause a considerable increase in the autophagosome load in various tissues [9,14,15]. The method involves starving the animals for 22 h to induce autophagy in the peripheral organs outside the central nervous system, and for more extended periods of time to induce autophagy in the mouse brain (figure 4*a*). We focused our analysis on ULK1, detected as a central node in the autophagy functional module by DOMINO (figure 2*b*), and compared the results with ATG14 and UVRAG since these genes exhibited increased expression in cells but were not selected by DOMINO. Unexpectedly, we could not detect changes in the mRNA levels of these genes in the brains of starved mice when compared with the levels in fed mice (figure 4*b–d*). This might be related to the mild autophagy response to fasting seen in the brain [48]. However, we cannot exclude the possibility that other secondary regulators affect the brain transcriptional programme during extended periods of fasting. By contrast, the results from the liver and skeletal muscles, tissues that are highly responsive to metabolic changes [49,50], support our active module analysis in that the levels of ULK mRNA were higher in the gastrocnemius muscle of fasted mice than in the muscles of fed mice (figure 4*b*). This increase in ULK mRNA was also observed in the liver of fasting animals (figure 4*d*). By contrast, the levels of ATG14 and UVRAG mRNA were less affected by starving than those of ULK1. A significant increase in ATG14 mRNA was detected only in the muscle of starved mice (figure 4*b*), with no significant changes in UVRAG mRNA in either muscle or liver of fasting animals (figure 4*b*,*d*).

### (d) Analysis of differential expression of autophagy genes in fat mice

We also examined whether a hypercaloric regimen could suppress autophagy gene expression in mice. For this purpose, we analysed RNA-seq data of tissues obtained from mice that were kept on a high-fat diet and compared them with control mice with a balanced food intake (electronic supplementary material, figures S2–S4). While we found significant differentially expressed autophagy genes in the brain, liver and skeletal muscle, only GABARAPL1 exceeded a 1.5-fold change. The high-fat diet increased GABARAPL1 expression in the skeletal muscle and suppressed GABARAPL1 expression in the liver (electronic supplementary material, figures S2–S4 and table S5).

## 4. Discussion

This study presents the results of an unbiased omics approach to the transcriptional response to starvation and high-fat diet in mammals. In recent years, omics analyses based on RNA-seq technology have been widely used to detect novel gene signalling networks. This may be accomplished by employing active module identification methods to discover connected sub-networks with an over-representation of highly active patterns [51,52]. Our recently developed DOMINO algorithm has a high rate of empirically confirmed calls and can overcome the common non-specificity issues obtained by many algorithms [30]. Importantly, our analysis indicated the activation of autophagy as a major component in the transcriptional response to starvation and revealed a high active module involving ULK1 sub-network genes, whose induction was verified in human cells and *in vivo* in mice. This suggests that targeting a small subset of the induced genes may achieve a selective effect (either inhibitory or stimulatory) on starvation-induced autophagy. Moreover, our study also suggests a tissue-specific effect of autophagy gene expression by fasting or hypercaloric regimens. For example, the reduced expression of GABARAPL1 in the liver of fat mice may be linked to disease, considering the role of autophagy in the metabolism of lipid droplets in hepatocytes [53].

Our data-capturing coding and non-coding transcriptomes provide a valuable resource for the identification of potential modifiers of autophagy and other important pathways affected by nutrient deprivation. This is exemplified in this study by the identification of differentially expressed lncRNAs, ATP6V0E2-AS1, RHPN1-AS1 and SLC7A11-AS1, which were recently associated with human diseases [41,54,55]. Further research will be needed to map and explore the entire spectrum of non-coding RNAs, such as circular RNAs and others, in response to fasting and fattening in mammals.

## Data Availability

Data are available at Dryad (https://doi.org/10.5061/dryad.1vhhmgqxp) [56]. Software is available at https://github.com/Shamir-Lab. Raw fastq files and processed data were uploaded to GEO (accession code GSE211066). Supplementary material is available online [57].
